# Supplementary material for: TSPO-PET reveals higher inflammation in white matter disrupted by paramagnetic rim lesions in multiple sclerosis
Source: Imaging Neurosci (Camb). 2026 Jan 22;4:IMAG.a.1107. doi: 10.1162/IMAG.a.1107 (PMC12828351; doi:10.1162/IMAG.a.1107)
Supplement: Supplementary Material [file IMAG.a.1107_supp.pdf]

Supplementary Information

Title: TSPO-PET Reveals Higher Inflammation in White Matter Disrupted by Paramagnetic Rim Lesions in Multiple Sclerosis

Running head: Higher inflammation due to paramagnetic rim lesions in MS

Authors: Ceren Tozlu PhD[1], Keith Jamison[1], Yeona Kang PhD [2], Sandra Hurtado Rua PhD [3], Ulrike W. Kaunzner MD PhD[4], Thanh Nguyen PhD [1], Amy Kuceyeski PhD [1*], Susan A. Gauthier DO, MPH [1,4*]

The average DVR from PKPET across the WM was lower in healthy controls (HC) compared to all MS patients. MS patients with PRLs showed a greater increase in DVR than those without PRLs, particularly within the deeper subcortical NAWM.

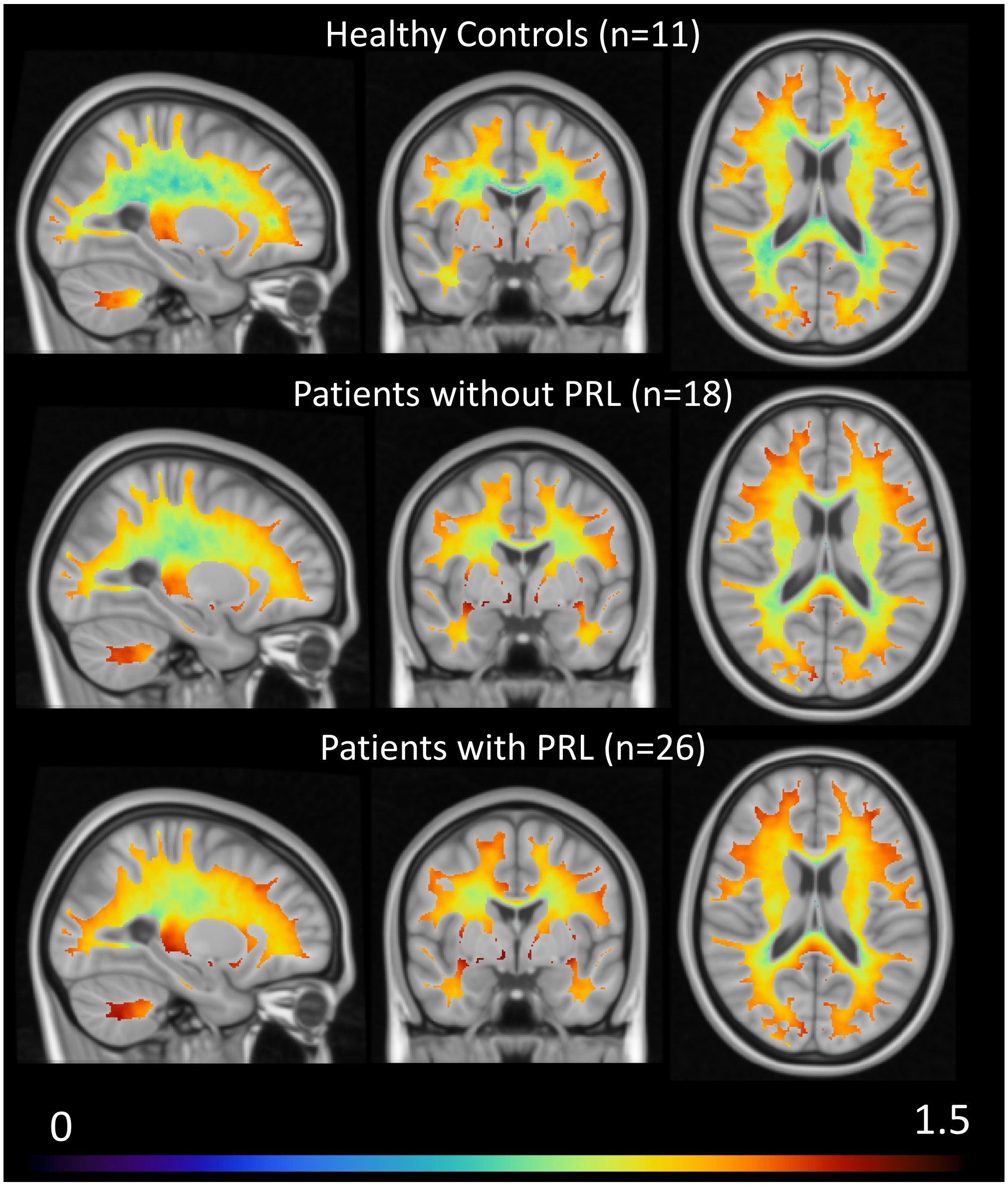

Supplementary Figure 1: The average DVR maps in the WM in healthy controls, patients with PRL, and patients without PRL. The voxels within 1 mm of proximity from ventricles and gray matter were excluded. The color bar represents the average DVR metrics.

Supplementary Figure 2 represents the T2 FLAIR lesion masks for all patients as well as the PRL and non-PRL lesion masks for the patients with at least one PRL (Panel A). The T2 FLAIR lesion burden was higher in patients with PRL compared to those without PRL. In patients with PRL, the PRL burden was higher compared to the non-PRL burden. Panel B shows that the disruption in the WM tracts due to T2 FLAIR lesions was also higher in patients with PRL compared to those without PRL. The posterior periventricular area is the most affected region due to both PRLs and non-PRLs.

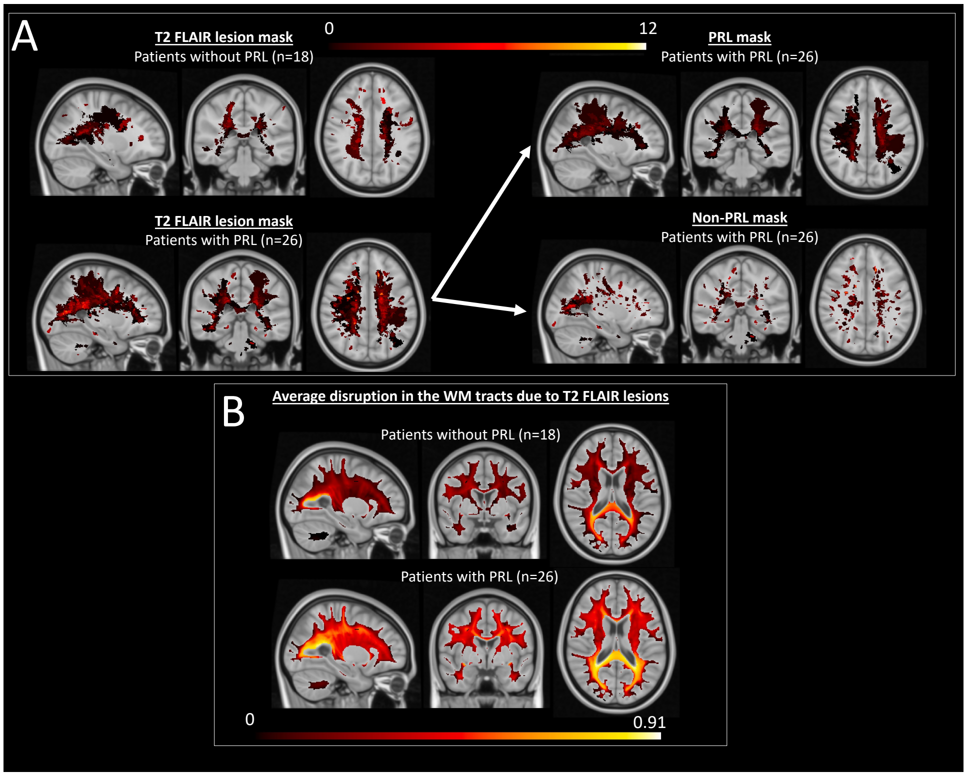

Supplementary Figure 2: (A) The left panel represents the T2 FLAIR lesion mask in patients with (N=26, figure on the top) and without PRL (N=18, figure on the bottom). The right panel represents the PRL and non-PRL masks in patients with PRL (N=26). (B) The average disruption in the WM tracts due to T2 FLAIR lesions in patients with (N=26, figure on the top) and without

PRL (N=18, figure on the bottom). The voxels within 1 mm of proximity from ventricles and GM were excluded from the figures.

Supplementary Figure 3 depicts the process that we used to create the WM mask, which was used then to measure the DVR in the normal-appearing WM. WM mask is created using the voxels that were present in at least 50% of the healthy controls.

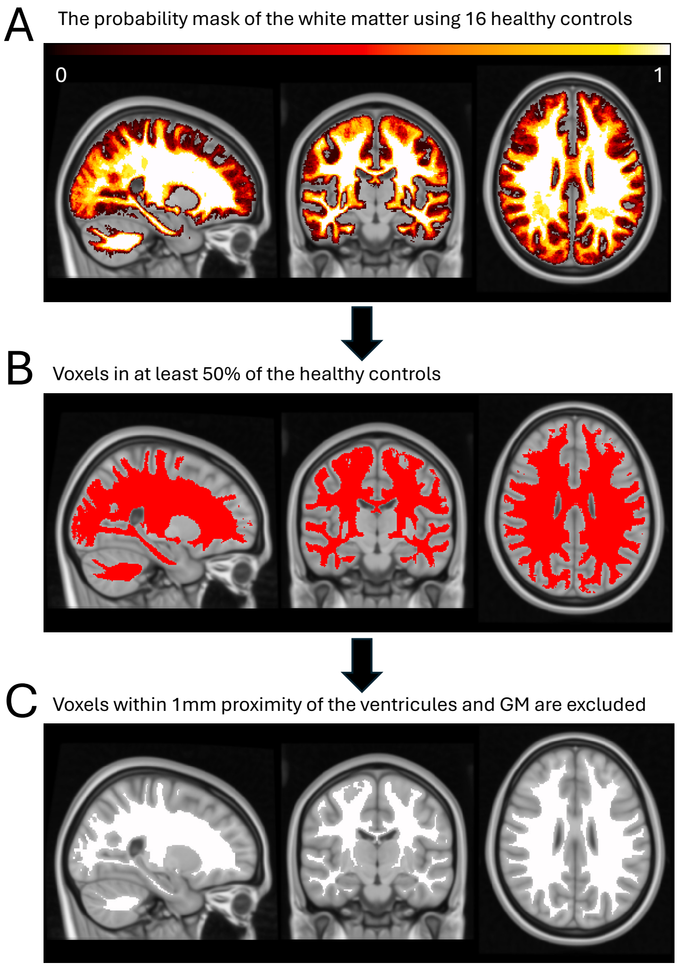

Supplementary Figure 3: (A) Probability mask generated using the WM segmentations in MNI space from 11 healthy controls. The probability mask shows the percentage of healthy controls with WM segmentation at each voxel, i.e. 0.8 indicates 80% of the healthy controls had WM on that voxel. (B) The thresholded version of the probability mask, including only WM voxels present

in at least 50% of the healthy controls. (C) Refined WM mask after excluding voxels within 1mm of the ventricles and gray matter. This final mask was used in our analyses.

The results from Figure 1 - that showed most voxel-wise DVR is higher in MS patients compared to controls as well as in patients with PRL compared to those without PRL - are consistent when ANCOVA is applied using age and sex as covariates.

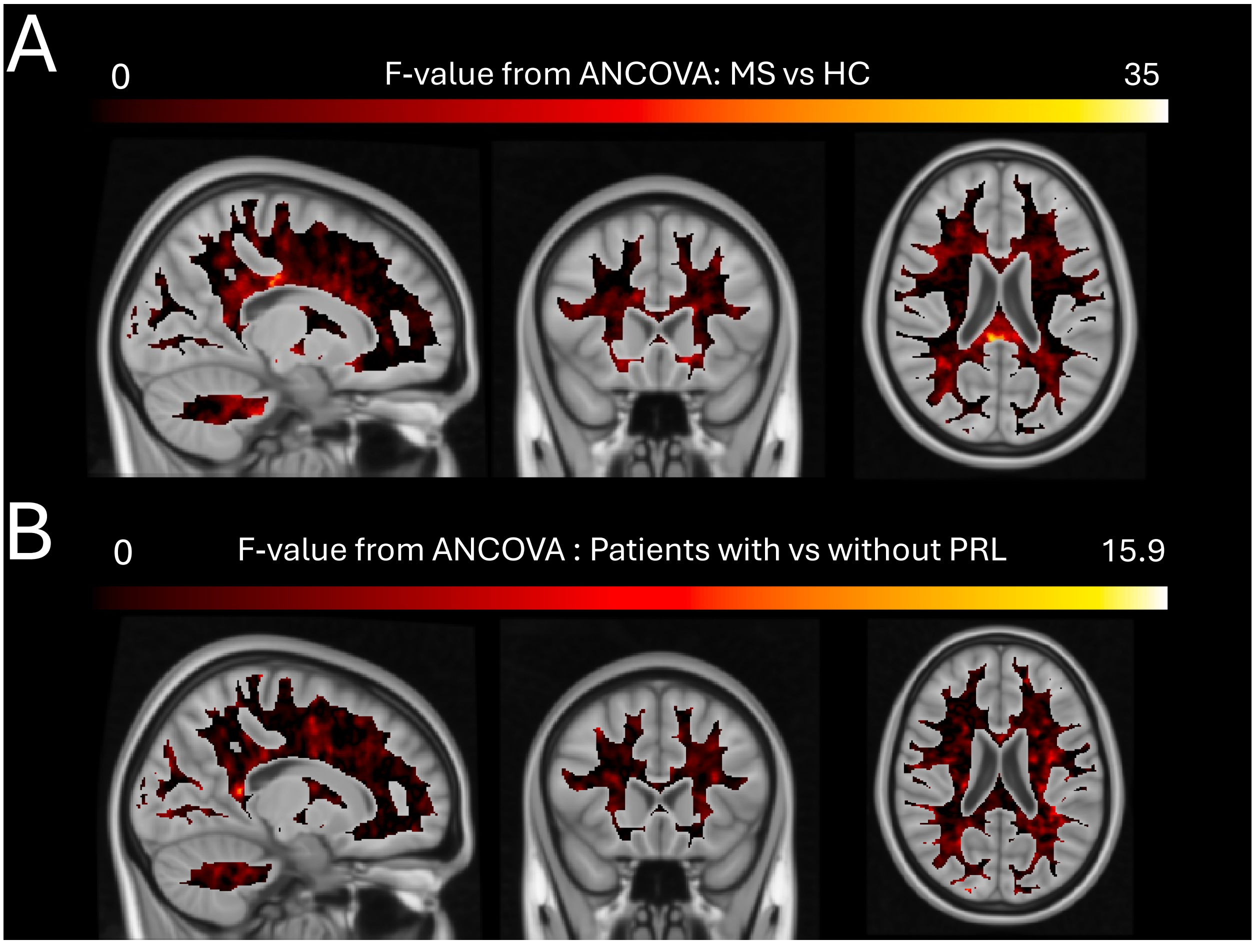

Supplementary Figure 4: (A) F-value from ANCOVA comparing voxel-wise DVR between MS patients and healthy controls. (B) F-value from ANCOVA comparing voxel-wise DVR between MS patients with vs without PRL. Age and sex were added as covariates in both ANCOVA analyses. The voxels within 1mm proximity to CSF and cortex were excluded.

In addition to measuring the average DVR metrics in WM tracts with low and high disruption, we also assessed the DVR in the WM tracts across a range of disruption levels from 0.1 to 0.9 in increments of 0.1 was also measured. The average z-scored DVR was significantly higher in MS patients with PRL compared to those without PRL in WM tracts with a disruption level between 0.7-0.8 (BH adjusted p-value=0.034) and between 0.8-0.9 (BH adjusted p-value=0.024). Additionally, among patients with PRL, the average of z-scored DVR was significantly higher in WM tracts disrupted by PRL compared to WM tracts disrupted by non-PRLs where the disruption level was between 0.7-0.8 (BH adjusted p-value = 0.031) and between 0.8-0.9 (BH adjusted p-value = 0.018).

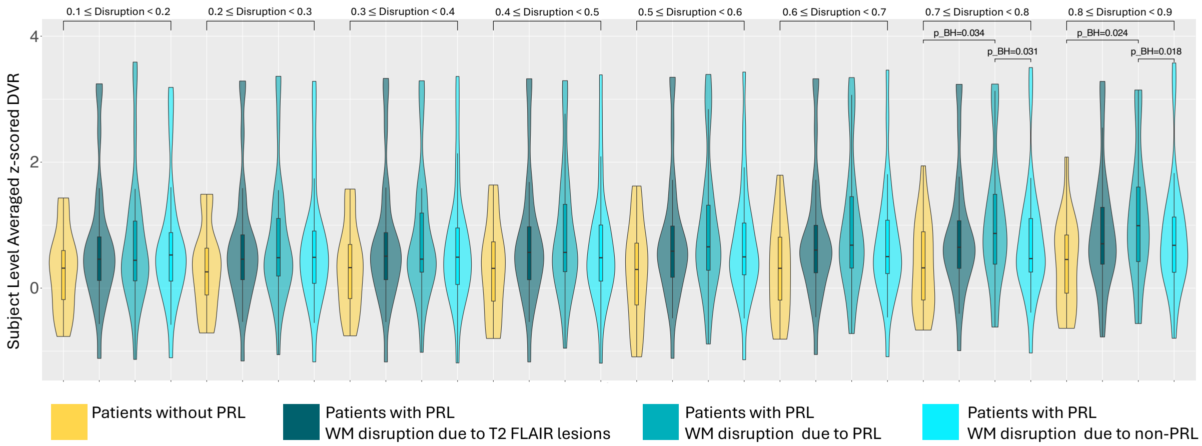

Supplementary Figure 5: The patient-level z-scored DVR metrics averaged across the WM tracts where the level of disconnection due to lesions (i.e. disruption in the WM tracts) was between 0.1 and 0.9. The voxel-wise z-scored DVR was created in the WM using the voxel-wise DVR in 11 healthy controls. Then, the patient-level averaged z-scored metrics were calculated for each MS patient separately. The disruption metrics were separated as disruption due to PRL and disruption due to non-PRL for patients with at least one PRL. The yellow color represents the patients without

PRL and the blue colors represent the patients with at least one PRL. The comparisons between the patients with vs without PRL were performed with ANCOVA where age, sex, and MS type were controlled. In patients with PRL, a paired t-test was used to compare the average of z-scored DVR in the WM tracts with varying levels of disruption due to PRLs vs non-PRLs. The BH-adjusted p-values (pBH) were presented in the figures.

In addition to examining the relationship between EDSS and the average DVR metrics in WM tracts with low and high disruption, we also investigated the association between EDSS and DVR across WM tracts with disruption levels ranging from 0.1 to 0.9 with increments of 0.1. There was a significant association between EDSS and the DVR in WM tracts disrupted between 0.8-0.9 in all MS patients (BH adjusted p-value=0.03) and the same trend was observed in patients with PRL (BH adjusted p-value=0.07).

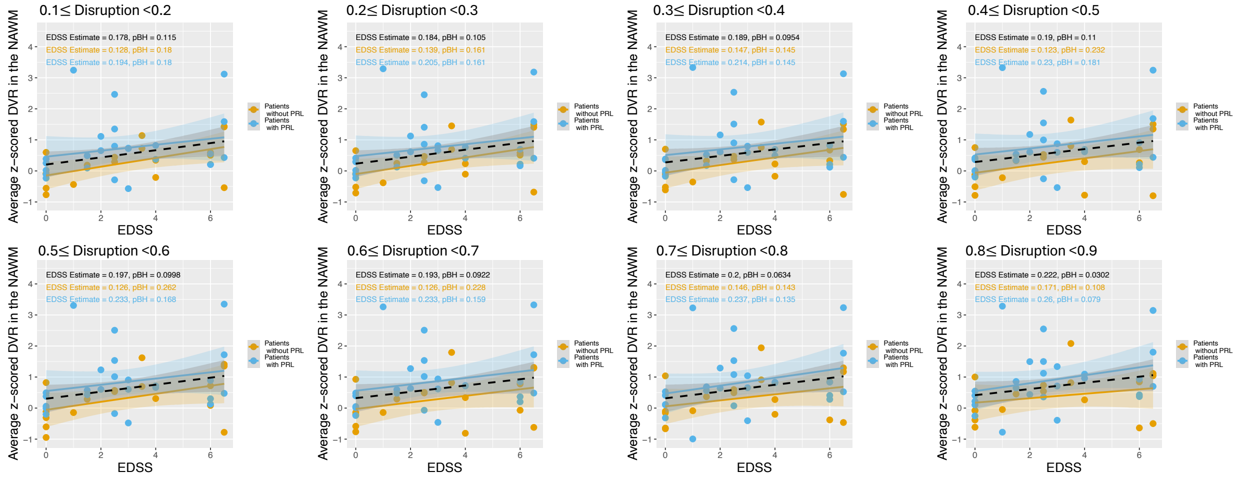

Supplementary Figure 6: The scatterplots of EDSS and subject-level averaged z-scored DVR for patients without PRL (orange color), patients with PRL (blue color), and all patients (black color). We computed the subject-level averaged z-scored DVR in the WM tracts with varying level of disruption in the WM tracts due to MS lesions, i.e. disruption in the WM tracts is between 0.1-0.9 with increments of 0.1. The estimates and the p-values associated with EDSS were calculated using a linear model where the output was the subject-level averaged z-scored DVR and the inputs were EDSS, age, sex, and MS type. The BH-adjusted p-values (pBH) were presented in the figures.
